# The lived experience of South African men having a premature baby

**DOI:** 10.4102/hsag.v29i0.2522

**Published:** 2024-04-30

**Authors:** Jonathan Nell, Kyle Jackson, Michelle Andipatin

**Affiliations:** 1 Department of Psychology, Faculty of Community and Health Sciences, University of the Western Cape, Cape Town, South Africa

**Keywords:** premature babies, fatherhood, masculinity, phenomenology, psychology, South Africa

## Abstract

**Background:**

Much has been written about fathers, fatherhood and premature babies. However, in the South African context, studies about the experiences of fathers having a premature baby are lacking.

**Aim:**

This study aimed to explore how South African fathers (*n* = 10) experience having a premature baby using a descriptive phenomenological approach.

**Setting:**

This research study was conducted online using various social media platforms such as WhatsApp, Google Meet and through telephonic conversations.

**Methods:**

A descriptive phenomenological approach that allowed for the distillation and elucidation of common core experiences among fathers who had a premature baby.

**Results:**

The findings demonstrated that the participants experienced intense fears regarding the survival and well-being of their children. They reported experiencing financial difficulties related to hospital bills and experienced being alienated by hospital institutions. Despite these reported barriers, these fathers were adamant in their resolve to support their children and partners during this challenging time.

**Conclusion:**

The experiences of fathers were riddled with fear, uncertainty, ambiguity and alienation, which placed them in very precarious situations when trying to navigate their role in a more sensitive and enlightened way. Having a premature infant calls into question the systems that men are positioned within as these systems to a large extent shape these events and how they are experienced.

**Contribution:**

This study is original as no other published studies seem to exist in South Africa that speaks to fathers’ lived experiences of having a premature baby.

## Introduction

Having a child is generally considered a joyous and revered experience as excitement and a greater level of life satisfaction are possible (Jackson & Andipatin 2019; Rizzo, Schiffrin & Liss 2013). This experience signifies a significant life event, as both men and women undergo a noteworthy identity change in the event of childbirth (Kowalenko, Mares & Newman 2012). The experience of integrating a child is exacerbated when having to be responsible for a premature baby, as stress, feelings of unworthiness, anxiety and a decrease in well-being are significant consequences of this event (Lindberg, Axelsson & Ohrling 2008; Vanier 2017). The aim of this study was to explore fathers’ lived experiences of having a premature baby in South Africa using a phenomenological approach.

## Fatherhood in South Africa

Traditionally, the role of fathers in South Africa has been perceived as being the protector and provider of the family and the mother as the nurturer and caregiver (Mavungu 2013; Ratele 2012). However, traditional constructions of fatherhood are contested in contemporary society and do not capture many fathers’ lived realities (eds. Van den Berg, Makusha & Ratele 2021). While the term father (biological or social) can be referred to as a man assuming responsibility for a child (Dayton et al. 2016) and fathering the everyday praxis concerning male parenting, the term fatherhood is meant to denote a larger discourse attempting to flesh out the abstract intricacies and constellations of being a father in society (Bermin & Long 2021; Handley 2019). Therefore, the term fatherhood is a hyperlink concept expanding the process and practice of being a father contextually and denotes what it means to be a father in certain parts of the world. More specifically, the construct can also be thought of as a phenomenon that is situated, relative and local with reference to South African men.

Research suggests that different conceptualisations of masculinity are challenging the seemingly traditional roles of fatherhood and, as a consequence, paternity and fatherhood alike (Amorim et al. 2017; Dayton et al. 2016; Enderstein & Boonzaier 2015; Lindberg et al. 2008; Tucker & Govender 2017). More fathers also lean towards assimilating the identity of becoming involved or hands-on fathers. This is evident in a recent Institute for Social and Health Sciences documentary depicting modern South African fathers’ (and mothers) perceptions of their roles within the family (Masculinity and Health Research Unit 2020). According to Ratele (2012), an involved father is characterised as a man who does not merely fulfil the traditional role of the father as a provider and disciplinarian but also closely and intimately cares for the well-being and development of his child(ren). Fathers are subsequently not constricted in their roles as providers and protectors but also have a psychologically shared experience of caring (Enderstein & Boonzaier 2015) despite the tension arising between the need to provide and be an involved caregiver (see Gatrell et al. 2015; Miller & Dermott 2015). This is especially significant as evidence suggests that fathers not only experience this caring postnatally but also experience it during the pregnancy (Makhanya 2018; Wong et al. 2016). In short, the perception of fathers as a bifurcated associate during pregnancy seems invalid (Amorim et al. 2017) relative to being a more involved and vicarious bearer of a child.

There has also been an increasing consideration of paternal mental health (as contrasted from maternal mental health) during and after the birth of a child (Baldwin & Bick 2017; Fletcher, Feeman & Garfield 2011; Jackson, Erasmus & Mabanga 2023). Mental health for fathers is important in child development (Pleck 2011; Kotelchuck & Lu 2017), as the positive effects thereof are considered equally important as and for maternal mental health (see Furaikh & Ganapathy 2016; Yu et al. 2011). Therefore, investigating the effects of having a premature baby is significant, as fathers are considered salient in the lives of their children.

## Premature babies

South Africa’s Western Cape government (WCG) defines prematurity as infants born less than 37 completed weeks of pregnancy, which also aligns with the World Health Organization’s (WHO’s) definition (WCG 2016; WHO 2018). Prematurity can be delineated into three subcategories, namely extremely preterm (< 28 weeks), preterm (28–32 weeks) and moderate to late preterm (32–37 weeks) (WHO 2018). Approximately 15 million babies are born prematurely every year, with this number increasing significantly in recent years with rates of between 5% and 18% (WHO 2018). Of these 15 million preterm births, it is estimated that 1 million result in death (WHO 2018). According to the WHO (2018), prematurity is also the leading cause of death among children under the age of 5 years old. In addition to these, the differences in the survival rates of premature babies are exponentially higher in developed, high-income countries compared to low-income countries like South Africa (WHO 2018). The burden of deaths among preterm babies is stark, which indicates a difference in the risk of being born prematurely and being born full term. This difference in premature mortality is also skewed relative to developing countries. For example, 90% of premature babies in developing countries die in the first few weeks compared to less than 10% in developed countries (WHO 2018). This skewness could be attributed to a range of systemic factors, such as income inequality, poverty and lack of access to adequate healthcare commonly found in developing countries (Leibbrandt, Finn & Woolard 2012). Therefore, the probability of these implications happening for babies born in developing contexts is likely high.

There are certain challenges that children face when being born prematurely. Highly probable mortality and developmental risks are inherent realities of being born prematurely (Davis & Burns 2001), with developmental risks ranging from learning disabilities, lifetime physical disability, auditory and/or visual impairments, among others (WHO 2018).

## Fatherhood and premature babies

A scoping review conducted by Jackson et al. (2023) found four prominent themes in the literature. The scoping review aimed to map the current literature of fathers experiencing high-risk pregnancies, which is intimately related to prematurity (Edwards et al. 2020). Fathers felt that healthcare staff and the hospital environment, which was often unwelcoming, provided them with limited communication, which contributed to their feeling neglected and near absolute exclusion regarding the state of their partners (Jackson et al. 2023). Personally, fathers felt exhausted, mentally drained and stressed, which echoes the emotional toll of having a partner with a high-risk pregnancy. These experiences also contribute to the ultimate role fathers see themselves playing and highlight major areas of support needed for fathers experiencing a high-risk pregnancy.

The capricious advent and relative emotional conflicts of having a baby born earlier than expected have a significant effect on fathers and are often categorised as a period of distress (Dadkhatehrani et al. 2018). The distress is compounded by increased periods of hospitalisation for the premature child (Straus, Bar & Stanger 2019), an internal and often deliberate denial or distortion of fathers’ own needs and emotional distress (which may be linked to hegemonic constructions of fatherhood) (Connell 2005; Mfecane 2016, 2018).

According to Dadkhatehrani et al. (2018), fathers experience various negative emotions, such as feelings of abandonment, helplessness, distrust in healthcare workers and high levels of anxiety. Fear regarding the uncertainty of survival of the hospitalised infant and the well-being of the mother is a consistent finding (Dadkhatehrani et al. 2018; Edwards et al. 2020; Vanier 2017). Feeling excluded from hospital processes and institutions emanates from the perception that men are not seen as part of the process of caring for their children. Fathers often report dissatisfaction with long waiting times, minimal information conveyed regarding the infant’s progress and disrespectful attitudes from professional healthcare workers (Dadkhatehrani et al. 2018). The experience of disconnection and alienation from the event, with the added anxiety regarding the survival of the infant and extra financial responsibilities, all characterise the stressful experience of fathers who have a premature infant.

Despite the literature indicating that fathers experience this event as stressful, there is a shortage of studies focusing on the experiences of South African fathers who had a premature baby. Considering that constructions of fatherhood in South Africa intersect with the range of macro-political, sociological and contextually specific factors such as unemployment and overburdened healthcare systems, this study contributes to the unique experience of involved fathers living in South Africa who struggle with the added adverse experience of having a premature baby.

## Research methods and design

### Research design

This study used a descriptive phenomenological research design, as proposed by Georgi et al. (2017). This Husserlian perspective, also known as transcendental phenomenology, concerns itself with pursuing different realities and not with establishing truth (Giorgi, Giorgi & Morley 2017).

### Participants and sampling

The sample consisted of 10 South African fathers recruited online from various parts of South Africa, with the majority living in the Western Cape (6), Gauteng (2), Mpumalanga (1) and the Eastern Cape (1). The majority of fathers identified as coloured (5), followed by white (3) and black (2). All fathers reported living with their children. All participants’ children were born relatively early, ranging from extremely preterm to moderately preterm (27–32 weeks). The mean age of the fathers was M = 37.7.

The main inclusion criteria to participate in the study were South African fathers who had a premature baby 6 months post the birth of their child. The classification of the preterm babies included all categories from 27 weeks to 37 weeks. Fathers also had to be 18 years and above, as this is the legal age for adults to provide consent, and participants needed to be able to speak English, Afrikaans or isiXhosa. However, all interviews were conducted in English, as preferred by all fathers. Saturation was reached by the 6th participant as no new data emerged from the interview process. Four more participants were recruited to confirm data saturation. Once initial contact was made, the researcher built rapport with the participants who met the inclusion criteria. Participants responded to an advert that was posted on social media platforms and proceeded to schedule online interviews with the first author. Interestingly, it was mostly mothers of babies born prematurely who made contact, and they indicated that the fathers would participate. The other 30% came from initial word-of-mouth and recommendations from these interviews.

### Research setting

Given the coronavirus disease 2019 (COVID-19) pandemic, this research study was conducted online using various social media platforms such as WhatsApp, Google Meet and through telephonic conversations. These platforms enabled the researcher to collect data digitally under the South African lockdown safety measures.

### Data collection

Data collection involved semi-structured interviews. The interview schedule was constructed in alignment with the aims and objectives and the relevant literature to enhance the trustworthiness of the study. Responses to these questions were recorded, with additional shorthand notes made during the interviews. The questions were also piloted with one participant to assess their relevance.

### Data analysis

Data analysis took the form of a descriptive phenomenological analysis using the guidelines proposed by Giorgi et al. (2017). The interviews were transcribed verbatim. Transcripts were reread and listened to give the researchers a basic sense of the data. Meaningful codes were delineated by grouping similar words and phrases to establish links and commonalities between what participants were communicating. The core essence or meaning of these linked categories characterised the universal experiences of fathers in the sample. Participants’ expressions were then intuited and reduced into psychologically meaningful categories to develop superordinate themes.

### Reflexivity

As a young person of colour who subjectively experienced his partner having a high-risk pregnancy, it was necessary to remain aware of my own thoughts, experiences and perceptions as both a man and father in the South African context. Reflexivity, as described by Palaganas et al. (2017), involves the examination of the researcher’s background, interests and perspectives as an overarching influence on and in relation to the research. Because of the inherent subjective nature of qualitative research, the perpetual awareness, assessment and reassessment of the researcher’s own experience with pregnancy was bracketed and set aside in the research process to establish transparency. A reflective journal was kept before and during the data collection and analysis process to write down any thoughts, emotions and perceptions. Although very difficult, this was an integral facet of being reflexive during the research process. Being a young male and father brought up ambivalent emotional responses in myself and in relation to the participants, who were mostly twice my senior. For myself, I felt the inherent anxieties and fears of having a baby, as this is a fragile and powerful process of bringing life into the world. Coexisting feelings of comfort and gratitude that my children are safe, and healthy were coupled with the anxieties felt when in conversation with the fathers. The conflicting feeling of being young relative to older participants and identifying with many of the experiences (anxieties and fears mentioned above) as a father were responses I had to be conscious about. Awareness of these responses helped to caution against biasing the results unconsciously through probing certain answers by asking unconscious leading questions that triggered these biased responses in myself. Thus, after every interview, these experiences were jotted down in a reflective journal. This also aided in dealing with these responses in subsequent interviews. For this reason, the researcher’s reflexivity was a constant feature.

### Ethical considerations

Ethics approval was obtained by the Bio-Medical Research Ethics Committee at the University of the Western Cape (BM20/8/12). All ethical principles and protocols were adhered to. Anonymity was assured by de-identifying all pertinent information. Participants were informed of their right to privacy and to withdraw at any stage. Electronic signatures for consent were requested from participants prior to the interview, and participants were subsequently informed about their role and rights in this study. The data were stored and protected on a password-protected computer. Participants were provided with details for counselling referral services. A short debriefing session after the interviews was held to check in with participants. Most fathers reported that talking about their experience was a positive sign of processing and integration. Some even suggested that they felt happy about sharing their experiences to raise awareness about the emotional rollercoaster of the event.

## Results

### My biggest concern was will she survive and be normal

#### Fear of child’s survival and developmental deficits

The birth of their children rapidly shifted fathers’ experiences to one that is characterised by intense fear and uncertainty about the well-being of their child as expressed by fathers in this study. Thabo expressed this more aptly:

‘… She was in hospital for 2 months, 2 and a half months. And then the child was not picking up weight. Her development was very slow. It was very stressful. And it was hard to see her at the time I was going there …’ (Participant 5, Thabo, Black Male, 28)

As a result of the stress, physical changes such as losing weight and a lack of sleep appeared common for all fathers. For most fathers, the fear was precipitated by witnessing their child as physically underdeveloped and suffering in hospital under life support. The excerpts from Riedewaan and Vuyo are presented below:

‘… I think my biggest concern was if she was going to be alive, because she was in a medically induced coma …’ (Participant 1, Riedewaan, Coloured Male, 27)

‘… Yoh biggest worry … I wanted her to live. But the biggest worry was the … because the drip and everything, shaving the head, the drip on the forehead; it was difficult to find a vain so they put it in the head. Actually, it was very painful on my daughter …’ (Participant 3, Vuyo, Black Male, 32)

A dark cloud of helplessness and powerlessness pervaded fathers’ experiences. Not being able to do anything, which is antithetical to notions of hegemonic masculinity, created feelings of worthlessness that goes against a central characteristic of protecting their children, which all fathers identified with. Riedewaan explores this further:

‘… Yeah and as a dad your job is to protect the children. The first thing that came to mind was “what could I have done”. For me that was the most stressful part. So I just felt helpless man, there wasn’t really much I could do, and that stressed me out …’ (Participant 1, Riedewaan, Coloured Male, 27)

Simultaneously, gnawing feelings of anxiety and fear around ‘normal development’ contributed to these as conflicting negative thoughts infiltrated and triggered their concerns of whether their children would live abled lives. This deep concern for their children to live ‘normal’ lives often led to perceptions that their children will not fit in socially and will require special attention. These experiences are expressed in the extract below:

‘Biggest worry, will he live a normal life? Will he live a normal life like any other child out there …’ (Participant 2, Justin, White Male, 30)

#### The financial side also kicks in

The emotional experience of fear about survival and anxiety around healthy development was accompanied by immediate engulfing omnipresent financial pressures, which was acutely felt in an already difficult time. This left fathers unprepared to deal with medical costs, especially given the lengthy period of intensive care in hospitals:

‘… And then the other financial side also kicks in. And that is now another stress. So, it’s just the fact that he must live and the finances that was the big thing because I think his story was a total of over three million rand …’ (Participant 6, Hannes, White Male, 29)

Exorbitant medical bills further evoked an overwhelming sense of anxiety, which exacerbated their internal emotional tug of war of helplessness and powerlessness and by extension disrupted the hegemony of the ‘provider’. This is captured in the statement below:

‘… I mean one day we asked for the bill for the day, and they gave us a stack of papers that size [gesturing with hands], and halfway through we just gave up because its … I think I stopped at R40 000 for the day. The number is just so big to think, and she was just in hospital for 3 months. So, yah, that takes over everything. I think when you the provider, the financial burden is huge …’ (Participant 4, Jeffery, Indian Male, 32)

## I had to be strong to support them

### Instinct to support

Resistance towards framing their role as imposed on them was directly in conflict with fathers’ ‘natural’ felt sense to support. Their motivation to support was reframed as having an internal source characterised by caring and a genuine felt concern about being there and supporting their children and families. This is asserted by Riedewaan:

‘… I don’t know if I have any thoughts or feelings about what was expected from me because I feel like what was instinctively there was to be there for my child and my partner …’ (Participant 1, Riedewaan, Coloured Male, 27)

Support was understood as being strong and resisting the possibility of ‘giving in’ to their difficult emotional experiences and not being seen as weak. Interestingly, fathers felt that ‘being strong’ by not expressing or communicating any feelings to their partners was important. Therefore, staying strong for the family often meant not expressing their own feelings and ‘doing what needs to be done’ and not revealing any distress as a man that is related to this experience. This is explained in the below extracts:

‘… To be strong for her, to be there for her. Despite me being a little scared. I mean, a man never shows his stuff [*emotions*] because you always have to be there for your wife. Because the wife has more stress and whatever. But the man also goes through this, but he never shows it …’ (Participant 2, Justin, White Male, 30)

‘… I had to be strong. Not just for me but for her as well. because she feeds off the energy that she gets like if we … if I am in a bad state and I am gonna be sad she’s gonna feel it because she is my child. She can sense when you in a bad state or when you happy. Whatever mood you in, they can sense it quick cause they feed from us as parents …’ (Participant 7, Marco, Coloured Male, 28)

The majority of fathers were able to make sense of their experience through the prism of staying strong for their partners and subsequently for the well-being of the baby. Staying strong entailed drawing on their faith in God and in the healthcare staff. The belief that the ‘mercy of God’ encouraged them to have a greater appreciation for their children and families. Coping in this way assisted with bearing the experience of uncertainty, worry and fear:

‘… Well we just stayed close to God, I mean it was just the important thing. And I think it just made our family stronger. And brought us closer to God because I mean we were in prayer a lot at that time. It is through the mercy of God that he made it …’ (Participant 6, Hannes, White Male, 29)

For many fathers, healthcare staff also relieved and contained some of the heavy emotional strain through their reassurance and perceived expertise. This is explained below:

‘… Yah, I kind of had faith and trusted the system and the people that were kind of dealing with the issue …’ (Participant 8, Ash, White Male, 31)

### Barriers to support

It is worth noting that not all fathers felt this way. A faction in the sample (4) felt that the healthcare system did not support their needs. Constant barriers to supporting their families, specifically pertaining to healthcare staff, hospital rules and regulations pervaded their experience. Disheartened fathers desperately explained that they should also be privy to information and without barriers to accessing their children:

‘… What I did have a lot of issues with was, in terms of institutions – they tend to, there’s all this conversation around fathers being active in their children’s lives …, and then institutions they tend to have this bias where everything that has to do with the child is the mother’s prerogative. I feel like institutions tend to disregard the father in those situations. And I feel like that’s a little discouraging man… like even leading up to the actual, you know before birth when we went to the ICU, they would make me wait outside, like I’m the child’s father and they make me wait outside when the mother goes in … Even in the hospitals, every time nurses and doctors speak about my daughter’s situation or give us an update or whatever, they tend to like, they will approach me and then they would ask where is the mother. I’m like you can tell me, I’m the father there’s nothing wrong with that. And they would insist on where’s the mommy. I’m like this is my child, you don’t have to wait for the mother, her father is here, you can tell me now. Why must you wait for the mother, you know what I’m saying … institutionally you get discredited and disregarded of the discussion around your child and what is happening around your child …’ (Participant 1, Riedewaan, Coloured Male, 27)

Barriers such as the COVID-19 lockdown regulations further prohibited fathers to access the hospitals contributing to feelings of rejection. Fathers reported being denied access to information about the status of their child and mother as some were not even able to know the gender of their child or whether the child or mother survived the birth experience:

‘… It was a bad experience due to this whole Covid pandemic … I didn’t even know what time the child came, and I didn’t even know what gender the child was. So, I tried to call the hospital as many times as I could, but they didn’t pick up their phones. But when I got through to them they didn’t want to tell me what gender the child was, cause they said because it’s over the phone and any random person can phone the hospital and say he is the father of the child. So, I had to wait a couple of days before I could find out the gender, or what gender the child is. And I didn’t even have a photo of my child cause the mother didn’t have a phone. So, I didn’t even know how the child look. The only time I saw the child was when the child was discharged from the hospital, and that was a week after she gave birth. So that was my traumatic experience of my second birth …’ (Participant 7, Marco, Coloured Male, 28)

Fathers often felt that they were not being supported. Incessant feelings of loneliness and fatigue because of work commitments, other parental responsibilities, being supportive and being present took away from their emotional experience in the moment. This further exacerbated feelings of exclusion in their attachment with their children. Oscillating between different places such as hospitals, workplaces and home for at least 3 months placed enormous strain on the physical and emotional well-being of fathers. This was essentially expressed as running on fumes, as adequate sleep and self-care eluded many of fathers’ experience:

‘… But I also think being a male, like we perceive males, you feel very alone because everybody is focused on the baby and the mother, and you have no support. So, I think that was, people would speak to you, but I would basically just be numb most of the time. I needed to be this pillar and you couldn’t sort of have emotions. So even attaching yourself to the child at the time, for me I couldn’t …’ (Participant 4, Jeffery, Indian Male, 32)

‘… You have to go work and you have to get over your tiredness. And you are the anchor in the family. So, you have to keep everything together and try to bring everyone through. I was worried and you are tired because you work every day. And if you get home at night, the eldest son was three, so all he knew was he came from the nanny and he gets in the car, and then we go to the hospital. He did his homework there and we drove there every night. We were there from six until eleven pm. So, you are tired, depleted, you do not know which way to go …’ (Participant 6, Hannes, White Male, 29)

As seen above, having a baby born prematurely does not only involve the ambivalent sense of losing a child and having to navigate the possibility of their children leading ‘normal lives’. This experience is also compounded by different forms of support (receiving institutional support and providing support) during this difficult time. However, despite the challenges faced by fathers, the majority of the fathers knew that their need for establishing a relationship with their children had receded into the background relative to ensuring the well-being of the child and mother.

### Stepping back

Fathers acknowledged that their babies needed their mother more than their fathers. Taking a backseat during the initial part of the experience was important as they afforded the mother time with the child:

‘… I was very … I had to take a back seat, because obviously they want the mother to be around quite a bit, and during those time they were very strict, they restricted the visiting times …’ (Participant 8, Ash, White Male, 31)

This was also accompanied by felt a sense of fear of hurting the child while exploring touch for the first time. Fathers described not wanting to worsen the condition of the baby by holding their children. This was a common experience and is explained in the below statement:

‘… I felt like initially I was a little bit afraid to like hold her and stuff because I just had this feeling, you know this very fragile view of her … like I said I was very paranoid because in the beginning I was even afraid to pick her up and hold her and stuff. So afraid that something would happen and stuff, you know what I mean?’ (Participant 1, Riedewaan, Coloured Male, 27)

The distance that fathers kept with their children was short-lived. As the child grew, fathers felt like they were becoming more involved through the experience of touching, changing nappies and feeding the child. For some, holding their baby felt like the beginning of their relationship with their child. Relationships were also characterised as starting off with immense fear, paranoia and possessiveness; however, witnessing their premature children grow and catching up to other children made fathers become gradually relaxed and less protective.

In an attempt to understand the essence of the experiences, one has to unpack the underlying emotions and how they coped, the perceived role fathers believed they should play under these circumstances and the support or lack thereof they experienced.

## Discussion

For South African fathers, having a premature baby proves to be challenging experience given the intrapersonal, interpersonal and larger systemic difficulties encountered in this context. Because of the uncertainty and trauma fathers experienced, a sense of fear was heightened and magnified. The physical manifestation of these fears is related to weight loss and an overwhelming sense of tiredness. Feelings of intense fear seemed to underpin the entire experience and are manifested in their fear for the child’s survival, financial fears and fears of not being able to hold it all together for the whole family, which also corroborates experiences in other contexts (Dadkhatehrani et al. 2018; Edwards et al. 2020; Vanier 2017).

Fathers often expressed feeling as if there was a dark cloud hanging over them, which contributed to the perceived stress about the mortality of their baby given how early they were born and the size of the baby. This culminated intense fears about their child’s survival and well-being, resulted in fathers losing weight and sleep deprivation precipitated by the tangible witnessing of life support machines.

Stress and anxiety regarding the anticipation of their children’s survival were often accompanied by their perceived inability to improve the situation. Feelings of helplessness and powerlessness pervaded their experiences as they felt there was nothing they could do. This also contributed to their feeling worthless. This narrative thus seemed to be located within a hegemonic masculinities ideology in which fathers are deemed to be protectors and ultimately define their roles (Handley 2019). This, the tension between feeling helpless and the role expectation, likely worsened the experience for fathers.

Financial strain experienced by these fathers was pervasive, with them reporting that they see themselves as the providers for their families, which also speaks to fathers’ perceived role in society (Mavungu 2013). This was embraced unquestioningly by all the participants in this study. Fears of how they would deal with enormous medical bills were experienced as extremely overwhelming. The external pressure of the ever-present and ever-increasing financial responsibilities exacerbated the fear of their child’s survival and well-being. The unexpected prematurity of their child’s birth meant that fathers were unprepared to cope with the extended, lengthy intensive care required that would directly impact their child’s survival and well-being. This forced fathers to be in a polarised state of helplessness and powerlessness.

It is worth noting that the presence of women in the workforce is obscured by hegemonic notions of being the provider and protector as black and coloured women have always worked in South Africa and beyond and become equal providers within the family space (Pinnock 2016). This means that fathers likely do not have to bear the financial burden alone. Thus, given the source of the financial burden placed on fathers having a premature baby, it does not necessarily translate to fathers having to be the sole bearers of this burden. Therefore, sampling fathers’ financial experience of having a premature baby may skew the veracity of how this burden may affect the family as a unit, reducing it to how it impacts the father alone. Despite fathers reporting this experience across this sample, and in the literature, this finding should be critically engaged with as the family as a whole may likely be affected and not just fathers alone.

A huge disjuncture emerged between the level of support needed and what was experienced. The ambivalence experienced thus related to the fact that fathers were expected to be involved fathers on the one hand, and on the other they felt that the institutional systems largely alienated them from their own experiences as well as those experiences relating to their partners and premature infants. The tension between being involved, being alienated and having to provide was also noted by Miller and Dermott (2015) and Gatrell et al. (2015).

Perceiving their role during this experience as being informed by external social commands was not accepted. It was reframed as having an internal source characterised by caring and genuine concern about being there for, caring for and supporting their children and families. Interestingly, fathers felt that being strong in the form of setting aside their feelings of helplessness and powerlessness and not expressing or communicating any feelings to their partners was key to being strong for their families, further echoing relentless hegemonic masculine roles.

Most fathers reported drawing strength from their faith and experience in the healthcare sector. For many fathers, healthcare staff also relieved some of the heavy emotional strain through their reassurance and expertise. The healthcare staff were instrumental in containing some of the difficult experiences mentioned above, making some fathers feel less stressed. A faction in the sample (4) felt differently. For some, there were constant barriers to supporting their families, specifically pertaining to healthcare staff and hospital rules and regulations. These gatekeeping regulations made fathers feel excluded, especially given the perceived widespread crisis fathers are aware of relative to not being present in their children’s lives (Van den Berg et al. 2021). Disheartened fathers desperately explained that fathers should also be privy to the information regarding their children in the hospital and should not encounter any inhibitions and barriers to information and access to their children.

Other regulations, such as the COVID-19 lockdown regulations, further alienated and strained the psychological well-being of some of the fathers going through this experience. Not having access to hospitals during the COVID-19 pandemic left fathers further alienated, as some of them were not privy to the gender of their child or whether the child and/or mother survived the experience or not. The lack of perceived support led to incessant feelings of loneliness and fatigue, a finding also noted by Dadkhatehrani et al. (2018). This occurred in the context of balancing multiple conflicting roles of work, home, parental obligations and being supportive to mom and child. Therefore, fathers could not invest in attaching or developing a relationship with the child. This physical and emotional toll in fulfilling multiple roles lead to a lack of self-care and sleep deprivation, which are precursors to possible undetected pathology.

These experiences are confirmed by Dadkhatehrani et al. (2018), who argue that fathers experience various negative emotions, such as feelings of abandonment and helplessness, which characterise their lack of assistance during this arduous experience. This is indicative of two key characteristic implications of having a premature baby. Firstly, fathers do not have enough support from healthcare staff during the emotional difficulties of having a baby born prematurely, as reported by fathers within the study. Secondly, despite these negative experiences, fathers persevere to provide support to the mother and child in the face of not being supported by healthcare staff as a means to care for the well-being of their partners and children.

One of the ways these fathers coped with the situation was by taking a back seat and choosing not to express their own vulnerability. In their view, they had to be strong for their partners and, in a sense, sacrifice their own well-being. There seemed to be an acknowledgement by fathers of the salience of the mother–infant bond immediately after the birth process and the importance thereof for the infant’s development (Holditch-Davis et al. 2002; Lindberg & Orhling 2008). For premature babies, this finding is particularly important as mothers are often involved in facilitating the survival of their babies. For example, aspects such as the mother–infant bond and the kangaroo method are crucial to the child’s survival. These aspects are largely enacted by mothers placing her at the centre of the foreground (WHO 2018).

Fathers felt they should not express any negative experiences, as it would put the well-being of the mother and child at risk. Therefore, not expressing these emotions served as a supportive strategy and was key for fathers having a premature baby. While not expressing emotional experiences to mothers was perceived as a protective factor for mothers, it can be considered a risk factor for fathers. This could be construed as prescribing hegemonic masculine ideals where men expressing their own authentic emotions is considered taboo (Dadkhatehrani et al. 2018; Pohlman 2005). However, these decisions are often made unconsciously and align with cultural norms of how men and women should enact their gender roles.

## Conclusion

The experiences of fathers were riddled with fear, uncertainty, ambiguity and alienation, which placed them in very precarious situations when trying to navigate their role in a more sensitive and enlightened way. Having a premature infant calls into question the systems that men are positioned within as these systems, to a large extent, shapes these events and how they are experienced. What was evident in the findings was how many health care practitioners, described by the participants as ‘gatekeepers’, kept many of them outside of their own experiences. The above highlights both the alienation that many of the participants experienced and how systems are constructed to perpetuate many gender inequities that many of us in society critique and struggle to overcome. Given the urgency and significance of the relationship between father involvement and child and maternal health, healthcare systems need to revise, adopt and implement progressive policies to facilitate and include fathers. Neglecting this crucial aspect may reinforce traditional notions, roles and practices of fatherhood and masculinity, which creates alienation and exclusion of South African fathers.

This study corroborated many of the findings in the literature on fathers having a premature baby. However, new findings were also uncovered, which speaks to the significance of this study. One of these findings relates to the COVID-19 pandemic and how the South African lockdown regulations excluded fathers from the birth process, which exacerbated the already arduous experience. Under ‘ordinary’ circumstances, many men felt alienated from the birthing experiences and the pandemic added to this burden. The estrangement that many of the participants expressed was palpable and extremely difficult to deal with. The implication of this is the heightened sense of powerlessness and helplessness experienced by fathers.

In short, the study’s findings point to the absolute necessity to focus on the inclusion, presence and participation of fathers in the pregnancy cycle of their partners. This sentiment gained traction and international recognition in 1994 following the International Conference on Population and Development (ICPD) recommendation, developed in Cairo (Egypt). An important recommendation from that conference proposed that ‘special efforts should be made to emphasise men’s shared responsibility and promote the effective involvement of men in responsible parenthood and sexual and reproductive behaviour’ (Soares et al. 2015:410).
